# Role of Membrane–Solute Affinity Interactions in Carbamazepine Rejection and Resistance to Organic Fouling by Nano-Engineered UF/PES Membranes

**DOI:** 10.3390/membranes13080744

**Published:** 2023-08-21

**Authors:** Oranso Themba Mahlangu, Mxolisi Machawe Motsa, Faisal Ibney Hai, Bhekie Brilliance Mamba

**Affiliations:** 1 Institute for Nanotechnology and Water Sustainability, College of Engineering, Science and Technology, University of South Africa, Florida Science Campus, Roodepoort 1709, South Africa; motsamm@unisa.ac.za (M.M.M.); mambabb@unisa.ac.za (B.B.M.); 2 Strategic Water Infrastructure Laboratory, School of Civil, Mining and Environmental Engineering, University of Wollongong, Wollongong, NSW 2522, Australia; faisal@uow.edu.au

**Keywords:** interaction energies, mixed-matrix membranes, organic compounds, nanoparticles, fouling prevention

## Abstract

In this study, polyethersulfone (PES) ultrafiltration (UF) membranes were modified with GO, Ag, ZnO, Ag-GO and ZnO-GO nanoparticles to improve carbamazepine removal and fouling prevention by making membrane surfaces more hydrophilic. The fabricated membranes were characterized for surface and cross-sectional morphology, surface roughness and zeta potential, as well as hydrophilicity, functional groups, surface tension parameters and water permeability Thereafter, the membranes were evaluated for their efficiency in removing MgSO_4_ and carbamazepine as well as antifouling properties. To understand the role of affinity interactions in rejection and fouling, membrane–solute adhesion energies (${∆G}_{slm}$) were quantified based on the Lifshitz–van der Waals/acid–base method. Unlike previous studies, which have generalized fouling prevention to be due to improvements in hydrophilicity upon adding nanoparticles, this work further explored the role of surface tension components on rejection and fouling prevention. The addition of nanoparticles improved membrane hydrophilicity (77–62°), water permeability (11.9–17.7 Lm^−2^ h^−1^ bar^−1^), mechanical strength (3.46–4.11 N/mm^2^), carbamazepine rejection (30–85%) and fouling prevention (60–23% flux decline). Rejection and antifouling properties increased as ${∆G}_{slm}$ became more repulsive (i.e., less negative). Membrane modification reduced irreversible fouling, and the fouled membranes were cleaned by flushing with water. Fouling related more to membrane electron donor components ($\gamma^{-}$), while the roles of electron acceptor ($\gamma^{+}$) and Lifshitz–van der Waals components ($\gamma^{LW}$) were less important. This work provides more insights into the role of affinity interactions in rejection and fouling and how rejection and fouling mechanisms change with nanoparticle addition.

## 1. Introduction

Water scarcity and the availability of potable drinking water have become a global concern. The little available water is often polluted with trace organic compounds, including pharmaceuticals, and these pollutants have been detected in different water sources across the globe [1,2,3,4,5]. The presence of trace organic contaminants in water poses a major health risk to humans; thus, their removal is imperative. Various water treatment techniques, such as activated sludge treatment, photocatalysis and electrocoagulation processes, have been applied for the removal of organic pollutants [6,7,8]. However, these processes do not achieve complete removal. Membrane treatment using ultrafiltration (UF), nanofiltration (NF) and reverse osmosis (RO) membranes has become a promising technique for the removal of organic contaminants in water, where the retention of organic compounds is controlled by membrane–solute interactions. This makes both membrane properties (e.g., molecular weight cut-off (MWCO), hydrophobicity and surface zeta potential) and solute properties (e.g., solute size and shape, charge, polarity and hydrophobicity) fundamental parameters in determining solute rejection [9]. Thus, electrostatic and non-electrostatic affinity interactions (e.g., hydrogen bonding and van der Waals interactions) are major membrane–solute interactions [10,11,12,13]. Based on size exclusion, solutes larger than the membrane effective pore size are well rejected, while smaller molecules pass through the membrane, thereby showing poor removal [14]. High solute rejection is attained when both the solute and membrane bear a similar charge due to electrostatic repulsions [15]. On the other hand, hydrophobic compounds show high initial rejection than hydrophilic compounds of similar molecular weight due to their adsorption on the membrane [13,15]. However, adsorption is a temporal phenomenon; after long-term filtration, the rejection of hydrophobic compounds declines due to their increase in concentration on the membrane surface (boundary layer) [16].

Membrane filtration offers advantages over other techniques, which include environmental friendliness, energy efficiency and ease of operation [17]. However, the application of membranes in water treatment is greatly hindered by fouling, which reduces membrane life-span and treatment efficiency [10]. Recent studies have focused on modifying membrane surface properties to improve hydrophilicity and reduce the prospects of membrane fouling [18,19]. One of the modification techniques is the incorporation of nanoparticles (e.g., graphene oxide (GO), ZnO, SiO_2_, WO_289_ and TiO_2_) into the membrane matrix [20,21,22,23]. In addition to fouling prevention, the resultant mixed-matrix membranes reject organic pollutants more than the control membrane without nanoparticles [10,20,23]. Some researchers have reported that the addition of nanoparticles into the membrane matrix increases the membrane mean pore radius [24,25]. This implies that the improved rejection of organic compounds smaller than the molecular weight cut-off (MWCO) of mixed-matrix membranes is through mechanisms other than size exclusion. Amongst these processes are solute–membrane affinity interactions whose effects on solute rejection have been overlooked or not well studied for membranes incorporated with nanoparticles. However, for commercial membranes, these effects have been widely investigated. For example, the transport of neutral organics was investigated in ion-exchange membranes and it was found that there was resistance for organics to partition into the membrane phase when the free interaction energies were greater than 0 [11]. Free interaction energies ($∆G_{i}$) influence solute rejection because they control the adsorption of organics on the membrane surface [13,26]. Previous studies have also shown that free energies of interactions between commercial membranes and solutes can be estimated from surface tension components through advanced contact angle measurements [10,11,27,28]. Using this approach, a good correlation was found between fouling and/or rejection and membrane–solute adhesion energies ($∆G_{slm}$).

For mixed-matrix membranes, previous studies have ascribed the improvement in rejection and antifouling properties to the membranes becoming more hydrophilic upon adding nanoparticles. However, no further investigation has been conducted on the contribution of electron donor ($\gamma^{-}$), electron acceptor ($\gamma^{+}$) and Lifshitz–van der Waals ($\gamma^{LW}$) components on solute rejection and antifouling properties of the nano-engineered membranes. Enhanced solute rejection properties of nano-engineered membranes can also be linked to improvement in water permeability properties of the membranes because some authors have demonstrated the dependency of rejection to flux [27,29,30]. However, extremely high fluxes and rejection cannot be achieved at the same time due to the tradeoff between the two [31]. Therefore, nanoparticles are added to attain satisfactory flux and rejection properties without sacrificing the other.

The aim of this study was to investigate the effects of modifying polyethersulfone (PES) membranes with graphene oxide (GO), zinc oxide (ZnO), silver (Ag), Ag-GO and ZnO-GO nanoparticles on the rejection of pharmaceuticals and membrane fouling propensity. These nanoparticles were selected because they have been widely used to enhance rejection, photocatalytic and antifouling properties of membranes due to their physical and chemical characteristics which include electronegative functional groups as well as optical and antimicrobial properties [10,25,32,33,34,35]. PES was chosen because it has excellent properties such as thermal stability, chemical resistance and strength. After synthesis and characterization, the rejection of carbamazepine as well as membrane fouling by sodium alginate was investigated. Carbamazepine was selected as a recalcitrant neutral organic compound so that the role of electrostatic interactions could be eliminated, while sodium alginate was chosen because membrane fouling by sodium alginate (representing extracellular polymeric substances in wastewater) has been widely investigated [36,37,38,39,40]. Many studies have shown poor removal of the recalcitrant carbamazepine compound in different water matrices [10,41,42]. Therefore, membrane modification to improve its removal is important. Unlike previous studies, changes in the membrane–solute affinity interactions upon adding nanoparticles were quantified and related to the rejection of carbamazepine [18,26,43]. Further, the study sought to understand better fouling mechanisms by relating membrane fouling parameters to specific membrane surface tension components (i.e., electron donor, electron acceptor and Lifshitz–van der Waals components).

Membrane–carbamazepine and membrane–sodium alginate adhesion energies (${∆G}_{slm}$) were estimated from contact angle measurements of solutes and membranes based on the Lifshitz–van der Waals and acid–base interaction energies [10,11,26,27]. The effects of incorporating different nanoparticles on the membrane surface tension parameters were investigated to identify the key components that change due to the modification. The findings were used to understand the improvements in flux, rejection and antifouling properties (or mechanisms) upon nanoparticle addition. This helped avoid the simple explanation of attributing enhancements in filtration properties to membranes becoming more hydrophilic—a common explanation given by most researchers. For the first time, this work investigates the correlation between ${∆G}_{slm}$ and membrane surface tension components ($\gamma^{-}$, $\gamma^{+}$ and $\gamma^{LW}$) with fouling parameters, namely, total fouling ratio (R_t_), reversible fouling ratio (R_r_) and irreversible fouling ratio (R_ir_), for nano-engineered membranes. This information gave an in-depth understanding of the underlying interactions that govern the mechanisms of fouling prevention by nano-engineered membranes.

## 2. Materials and Methods

### 2.1. Synthesis of Nanoparticles

#### 2.1.1. Synthesis of Graphene Oxide

Graphene oxide (GO) was synthesized from graphite (<45 μm, ≥99.99% trace metals basis, Merck, Johannesburg, South Africa) based on our previous approach, which was adopted from the Hummers method [10]. Briefly, 6 g of sodium nitrate (ACS reagent, >99.0% NaNO_3_, Merck, South Africa) was added to a 1 L glass beaker containing 8 g graphite grains. The beaker was safely secured in an ice bath, and this was followed by addition of 270 mL sulfuric acid (ACS reagent, 95% H_2_SO_4_, Merck, South Africa). The mixture was stirred vigorously, and 36 g potassium permanganate (ACS reagent, >99.0% KMNO_4_, Merck, South Africa) was slowly added over 60 min. After vigorous stirring for an additional 120 min, the beaker was removed from the ice bath and stirred under room temperature for 120 h. This was followed by slow addition of 400 mL of 5% H_2_SO_4_ over a period of 60 min, and the solution was heated gradually to 98 °C over a period of 60 min (Heating and Drying oven; model: DHG-9023A, Everich, Hangzhou, China). The suspension was stirred for 120 min at constant temperature (98 °C) and cooled down to room temperature. Once at room temperature, 80 mL of 30 wt% hydrogen peroxide (30% *w*/*w* H_2_O_2_, Merck, South Africa) was added to react with excess KMNO_4_, followed by stirring for 120 min. The suspension was centrifuged at 4000 RPM for 20 min (Sigma 3-16 P, Sigma, Johannesburg, South Africa) to separate the GO. This was followed by repeated washing with 5% HCl (ACS reagent, 37% HCl, Merck, South Africa) and deionized water (Millipore Corp, Temecula, CA, USA). The GO was dried in an oven at 60 °C for 18 h (Heating and Drying oven; model: DHG-9023A, Everich, China).

#### 2.1.2. Synthesis of Silver (Ag) and Ag-GO Nanoparticles

The preparation of Ag-GO nanoparticles began with the synthesis of Ag nanoparticles as follows: 6 g of 99.99% trace metal basis silver acetate (Merck, South Africa) was placed in a glass beaker and 300 mL deionized water was added, followed by stirring for 60 min. Sodium borohydride (0.5 M NaBH_4_, ACS reagent, >98%, Merck, South Africa) was added dropwise, resulting in color change, and the mixture was stirred for 30 min. The suspension was filtered, washed with deionized water several times and dried overnight at 90 °C (Heating and Drying oven; model: DHG-9023A, Everich, China).

For the synthesis of Ag-GO nanoparticles, 1.5 g of the previously synthesized GO and 1.5 g of Ag nanoparticles were individually suspended in deionized water and sonicated for 30 min. The two suspensions were mixed and ultrasonicated for an additional 30 min (Ultrasonic S60H, Elmasonic, Singen am Hohentwiel, Germany). The mixture was stirred for 120 min, and the pH was adjusted using ammonium hydroxide (ACS reagent, 28.0–30.0% NH_3_ basis, NH_4_OH, Merck, South Africa) to pH 7. After pH adjustment, the mixture was kept in an oven at 90 °C overnight. This was followed by washing several times with deionized water and centrifugation at 4000 RPM for 30 min (Sigma 3-16 P, Sigma, South Africa). The Ag-GO nanoparticles were dried at 70 °C overnight, calcined at 500 °C (Lenton Furnaces, Neuhausen, Germany) and kept for further characterization and use.

#### 2.1.3. Synthesis of Zinc Oxide (ZnO) and ZnO-GO Nanoparticles

ZnO nanoparticles were synthesized from reagent-grade, >98% zinc chloride (ZnCl2, Merck, South Africa) and NH4OH (CS reagent, 28.0–30.0% NH3 basis, Merck, South Africa). ZnCl_2_ (0.68 g) was suspended in deionized water, and the pH was adjusted to pH 7 using 25 wt% NH_4_OH. The suspension was kept static in an oven at 90 °C for 9 h (Heating and Drying oven; model: DHG-9023A, Everich, China), after which it was left to cool to room temperature. This was followed by filtration and washing with ethanol (ACS reagent, Merck, South Africa) and water. The nanoparticles were then dried in an oven at 80 °C for 24 h.

ZnO-GO nanoparticles were synthesized by first dispensing 0.5 g of the previously prepared GO nanoparticles in deionized water and sonicated for 30 min (Ultrasonic S60H, Elmasonic, Germany). ZnCl_2_ solution (0.5 mM) was then added to the solution dropwise, followed by ultrasonication for an additional 30 min (Ultrasonic S60H, Elmasonic, Germany). The mixture was stirred for 120 min, after which the pH was adjusted, using NH_4_OH, to pH 7. The mixture was kept static at 90 °C for 9 h and later centrifuged (Sigma 3–16 P, Sigma Aldrich, South Africa) for 30 min at 4000 RPM. The supernatant was decanted, while the residue was dried in an oven at 80 °C overnight. This was followed by calcination at 500 °C for 2.5 h (Lenton Furnaces, Germany).

### 2.2. Characterization of the Nanoparticles

#### 2.2.1. SEM Micrographs and EDS Spectroscopy

Successful synthesis of the nanoparticles was confirmed by surface imaging and analysis of chemical composition using a scanning electron microscope (SEM, Jeol JSM IT300, Tokyo, Japan). The SEM was coupled with an energy-dispersive spectroscopy (EDS) analyzer (EDS, Jeol JSM IT300, Tokyo, Japan). Prior to analysis, the powdered samples were coated with 5 nm gold in an SCD 005 Cool Sputter Coater (BalTec, Lübeck, Germany) at a current of 25 µA, which was applied for 50 s. Surface images were recorded at irradiation beam of 10 kV, while EDS spectra were recorded under an irradiation beam of 40 kV. Surface imaging and identification of chemical composition were important to establish the morphology of the nanoparticles, as well as to confirm chemical composition of the nanomaterials.

#### 2.2.2. Particle Size and Zeta Potential

The nanoparticles were characterized for surface zeta potential and particle size using a Malvern Zetasizer Nano series (Malvern, UK). Size and zeta potential measurements were performed at neutral pH. The background electrolyte was 10 mM KCl (ACS reagent >99.9%, Merck, South Africa). Zeta potential measurements were related to the electrophoretic mobility (EM) of the nanoparticles according to the Helmholtz–Smoluchowski equation (Equation (1)), which considers zeta potential (ζ, mV), Henry’s function (*f* (*ka*)), permittivity of water (ε, C^2^N^−1^ m^−2^) and electrical viscosity (μ, Pa.s)

(1) $$EM=\frac{2\varepsilon \zeta f(ka)}{3\mu }$$

The sizes of the nanoparticles were determined by means of dynamic light scattering (DLS) techniques under background electrolyte of 10 mM KCl.

### 2.3. Fabrication of Pristine and Nanocomposite Membranes

#### 2.3.1. Preparation of Casting Solutions

Casting solutions with and without nanoparticles were prepared by first dissolving polyethersulfone (Mw 35 kDa PES, Solvay, Belgium) in N-Methyl-2-pyrrolidone or NMP (ACS reagent, >99%, Merck, South Africa) under room temperature. Each casting solution contained 22 wt% PES (Table 1). For preparation of casting solutions with nanoparticles, desired amounts of the nanoparticles were individually dispersed in a little bit of NMP solvent (approximately 5 mL) and ultrasonicated (Ultrasonic S60H, Elmasonic, Germany) for 30 min. The suspensions were then added to the previously prepared PES casting solutions (except for the casting solution prepared for the pristine membrane) and stirred for 1 h to mix thoroughly. All casting solutions were kept in the dark overnight to ensure complete removal of air bubbles prior to membrane casting. The nanoparticles were added at 0.2 wt% based on our previous works, where loadings >0.2 wt% resulted in low fluxes (or no further improvement in membrane properties) due to nanoparticle agglomeration and blocking of membrane pores [10], and this has also been observed in other works [44,45,46].

#### 2.3.2. Fabrication of Pristine and Nanocomposite Membranes

The conventional non-solvent phase inversion technique was used to fabricate the pristine and nanocomposite membranes. After preparing and degassing the casting solutions, the membranes were cast on a glass plate using a casting knife (Elcometer 3530, Elcometer, Liege, Belgium). The casting knife gap clearance was first adjusted to 200 μm to control the membrane thickness, and the individual casting solutions were spread on a dry glass plate. The glass plate was then transferred into a water bath to initiate coagulation at room temperature for 30 min, after which the membranes were placed in plastic bags with water and kept in the refrigerator at 5 °C overnight to complete the phase inversion process and prevent bacterial growth. The membranes were named and marked based on the nanoparticles added (Table 1). For example, a membrane with graphene oxide was named GO membrane.

### 2.4. Characterization of the Membranes

Chemical composition and/or functionality of the membranes (after drying overnight in a desiccator) was characterized by attenuated total reflectance Fourier transform infrared (ATR-FTIR) spectroscopy (Perkin Elmer, FT-IR 100, PerkinElmer, Inc., Shelton, CT, USA). Prior to conducting FTIR analysis, a background scan was performed, and all scans were performed from 4000 cm^−1^ to 500 cm^−1^ with a resolution of 4 cm^−1^ and 4 scans per sample.

A Raman spectrometer (Alpha 300A, WITec, GmbH, Ulm, Germany) was used to characterize the membranes for their functionality. The membrane samples were individually mounted on separate glass slides, which were then placed under an Olympus BX51 microscope. Raman measurements were conducted using a 750 nm laser after focusing the laser beam on the measured sample. The grating and spectral center were adjusted to 300 g/mm and 1800 cm^−1^, respectively. A total of 30 accumulations were recorded for an integration time of 30 s.

Contact angles were measured for the previously dried membrane coupons using a contact angle analyzer or goniometer (DSA30E, Kruss GmbH, Hamburg, Germany). The measurements were based on the sessile drop technique, where three types of liquids—Milli-Q water, glycerol (ReagentPlus, >99%, Merck, South Africa) and diiodomethane (ReagentPlus, >99%, Merck, South Africa)—were used for contact angle measurements. These liquids have well-characterized surface tension parameters. For each membrane type, a minimum of 10 drops per liquid was placed on the surface of each membrane. A microliter syringe was used to accurately deposit 5 µL of the desired liquid. All measurements were carried out at room temperature, and images of the water contact angle were recorded. The measured contact angles were then used to quantify surface tension components based on the Lifshitz–van der Waals/acid–base approach, as explained in our previous works [10,27].

Surface and cross-sectional micrographs of the membranes were obtained using a scanning electron microscope (Jeol JSM IT300, Tokyo, Japan) at an irradiation beam of 10 kV. The membranes were first dried in desiccators for at least 24 h before SEM analysis. For cross-sectional imaging, the dried membranes were immersed in liquid nitrogen, frozen and broken. SEM analysis was conducted after coating the membrane with gold at a current of 25 μA for 50 s using an SCD 005 Cool Sputter Coater (Bal-Tec, Untersiemau, Germany).

A WITec Alpha 300 atomic force microscope or AFM (WITec, GmbH, Erfurt, Germany) was utilized to obtain AFM micrographs of the membranes. Measurements were conducted on previously dried membranes in non-contact mode using reflex-coated FM (AC), 2.8 N/m, 75 kHz AFM Arrow cantilevers. Surface roughness parameters of the membranes (S_a_ and S_q_) were quantified using Project 5 (WITec, GmbH, Erfurt, Germany) after doing a background correction.

A SurPASS Electrokinetic Analyzer (Anton Paar, GmbH, Graz, Austria) was used to measure streaming potentials of the membranes. Measurements were performed at pH 7 and 10 mM KCl background electrolyte. The tangential mode of analysis was used at a pressure of 200 mbar and gap height of 105 μm. The zeta potentials (ζ) were then estimated according to the Helmholtz–Smoluchowski equation (Equation (2)), which considers the measured streaming potential (Δ*V*, mV), electrolyte viscosity (μ, Pa.s) applied pressure (Δ*P*, Pa), electrolyte conductivity (*δ*, µS/cm) and permittivity of water (ε, C^2^N^−1^ m^−2^).

(2) $$\zeta =\frac{∆V\mu \delta }{∆P\varepsilon }$$

The porosity of the fabricated membranes was quantified through the dry–wet approach. Briefly, the membranes were cut into small portions (4.2 × 8.7 cm^2^), and permeate flow (Q) was determined at 4 bar using a crossflow (see Section 2.5). Flow measurements were conducted after membrane compaction at 6 bar until stable fluxes. After flow measurements, the membranes were removed from the filtration cells, and the wet masses were measured after wiping off superficial water from the membrane surfaces. Thereafter, the membranes were dried at 45 °C overnight, and the dry weights were measured after the membranes were allowed to cool to room temperature. Membrane porosity (η) was calculated using Equation (3), which considers the weight of the wet membrane (*W_w_*, g), the weight of the dry membrane (*W_d_*, g), membrane area (*A*, cm^2^), membrane thickness (σ, cm) and density of water (ρω, g.cm^−3^).

(3) $$\eta \left(\%\right)=[\frac{W_{w}-W_{d}}{\rho \omega A\sigma }]\times 100$$

The mean pore radius (*r_m_*) was calculated using the Guerout–Elford–Ferry equation (Equation (4)), which incorporates membrane porosity (η), water viscosity (μ, 8.9 × 10^−4^ Pa.s), membrane thickness (σ, m), permeate flow (Q, m^3^ s^−1^), effective membrane area (A, m^2^) and applied pressure (ΔP, Pa).

(4) $$r_{m}=\frac{\sqrt{\left(2.9-1.75\eta \right)8\mu \sigma Q}}{\eta .A.∆P}$$

### 2.5. Filtration Experiment Protocol

#### 2.5.1. Filtration Setup

**Figure 1:**
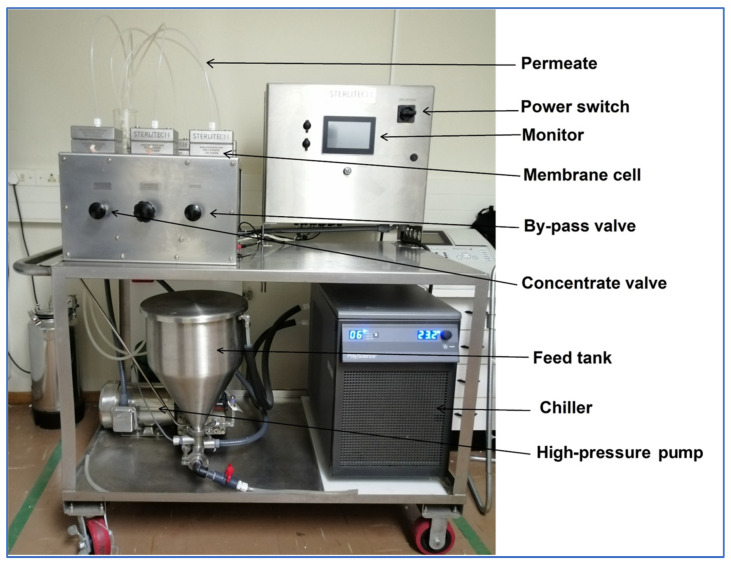
Crossflow filtration unit used for flux, rejection and fouling experiments.

Filtration experiments were carried out using a custom-made cross-flow filtration setup (Figure 1) with the following channel dimensions: channel width of 4.2 cm, channel length of 8.7 cm and channel height of 0.1 cm. The system consisted of 6 membrane cells that could be operated independently. A high-pressure pump (Hydra-Cell; Wanner Engineering, Minneapolis, MN, USA) was used to deliver feed water into the membrane cells from a 20 L stainless steel feed tank.

For all the membranes, filtration experiments were conducted at initial permeate flux of 40 L/m^2^ h and crossflow velocity of 0.2 m/s. Membrane water flux ($J_{w}$, L/m^2^ h)) was estimated from Equation (5) based on the volume of permeate collected (*V*, L) at specific time (*t*, s) and membrane area (*A*, m^2^). From water flux, the membrane resistance ($R_{m}$) was calculated from knowledge of applied pressure (Δ*P*, Pa) and viscosity of water (μ, Pa.s).

(5) $$J_{w}=\frac{V}{At}=\frac{∆P}{\mu R_{m}}$$

The membrane pure water permeability ($L_{p}$) was calculated using Equation (6):

(6) $$L_{p}=\frac{J_{w}}{∆P}$$

Due to the differences in membrane permeability, the applied pressure was varied for the different membranes to achieve the desired initial permeate flux of 40 L/m^2^ h. The applied pressure was controlled by adjusting the bypass and concentrate valves; the feed pressure was measured using a pressure gauge (ERIKS, Antwerp, Belgium). All filtration experiments were conducted in recycling mode where the permeate and concentrate were returned into the feed tank.

Prior to conducting filtration experiments, the membranes were compacted at 6 bar for 3 h, and membrane fluxes were measured at different applied pressures. Flux measurements were followed by determination of the rejection of 2000 mg/L MgSO_4_ (ACS reagent, >98% MgSO_4_.7H_2_O, Merck, South Africa) and 5 mg/L carbamazepine (powder, Merk, South Africa). The solutes were added to the feed to achieve the desired concentrations, and the system was allowed to equilibrate for 6 h before collecting samples (feed and permeate) for analysis. A longer equilibration time was used to ensure that carbamazepine rejection was not biased due to temporal adsorption of the compound by the membrane.

#### 2.5.2. Assessment of Membrane Rejection Properties

Carbamazepine rejection was monitored by measuring the total organic carbon (TOC Fusion, Teledyne Tekmar, Mason, OH, USA), while the concentration of salts was examined by analyzing electrical conductivity (Consort C6010 conductivity meter, Consort, Turnhout, Belgium). Rejection (R) was calculated using Equation (7), where $C_{p}$ and $C_{f}$ are solute concentrations in the permeate and feed, respectively.

(7) $$R\left(\%\right)=[1-\frac{C_{p}}{C_{f}}]\times 100$$

The TOC analyzer had a limit of detection of 0.1 mg/L; thus, 5 mg/L carbamazepine was used to enable quantification at 98% carbamazepine rejection.

#### 2.5.3. Investigation of Membrane Antifouling Properties

Sodium alginate (medium viscosity, Merck, South Africa) was selected as model foulant representing natural organic matter (NOM). Sodium alginate was characterized for charge and size at pH 7 in a background electrolyte of 10 mM KCl. A Zetasizer nano series (Malvern Instruments, Malvern, UK) was used to measure both foulant charge and size, where the charge was estimated from the electrophoretic mobility, while size measurements were based on dynamic light scattering techniques. This was followed by investigating membrane fouling behavior by 20 mg/L sodium alginate at pH 7 and 10 mM NaCl (ACS reagent > 99.9% NaCl, Merck, South Africa) as background electrolyte. Prior to fouling, the membranes were compacted at 6 bar until stable fluxes were obtained, and membrane fouling was conducted at an initial flux of 40 L/m^2^ h and cross-flow velocity of 0.2 m/s.

Initial membrane fouling is controlled by membrane–foulant affinity interactions. Thereafter, fouling depends on the interactions between foulants deposited on the membrane surface and those coming from the feed. Membrane modification aims to minimize initial fouling and/or irreversible fouling. Therefore, in this study, fouling was conducted for 3 h to investigate if the addition of nanoparticles could limit the deposition of sodium alginate on the membrane surface and permeate flux was monitored at selected time intervals. Antifouling properties were quantified by calculating the flux recovery ratio (*FRR*), the total flux decline ratio (*R_t_*), as well as irreversible (*R_ir_*) and reversible (*R_r_*) fouling ratios. After fouling studies, the different fouling ratios were determined by cleaning the membranes with deionized water. This was followed by re-measuring pure water flux, and the different fouling ratios were estimated according to Equations (8)–(11).

(8) $$FRR\;(\%)=[\frac{J_{w2}}{J_{w1}}]\times 100$$

(9) $$R_{t}(\%)=[1-\frac{J_{f}}{J_{w1}}]\times 100$$

(10) $$R_{r}\;(\%)=[\frac{J_{w2}-J_{f}}{J_{w1}}]\times 100$$

(11) $$R_{ir}\;(\%)=[\frac{J_{w1}-J_{w2}}{J_{w1}}]\times 100$$

where ($J_{w1}$) is pure water flux prior to fouling, ($J_{w2}$) is pure water flux after fouling and ($J_{f}$) is flux of the foulant solution (in this case, at 3 h of fouling with sodium alginate). The deposition of foulants on the membrane surface was confirmed by recording SEM images as well as microscope images using Spotlight 400 FTIR Imaging system (PerkinElmer, Inc., Waltham, MA, USA).

### 2.6. Calculation of Free Energies of Interactions

The estimation of free energies of interactions was based on our previous works [10,27]. Lifshitz–van der Waals component ($\gamma^{LW}$), electron acceptor ($\gamma^{+}$), electron donor ($\gamma^{-}$) were calculated from the measured contact angles according to Equation (12).

(12) $$\left(1+\frac{Cos\theta }{r}\right)\gamma_{s}^{TOT}=2(\sqrt{\gamma_{s}^{LW}\gamma_{l}^{LW}}+\sqrt{\gamma_{s}^{+}\gamma_{l}^{-}}+\sqrt{\gamma_{s}^{-}\gamma_{l}^{+}})$$

where r represents an increase in surface area due to membrane roughness; subscripts s and l are the solid surface and test liquid, respectively; and $\gamma^{+}$ and $\gamma^{-}$ represent the Lewis acid–base components, respectively. The Lewis acid–base components ($\gamma^{AB}$) can be estimated from $\gamma^{AB}=2\sqrt{\gamma^{+}\gamma^{-}}$.

Contact angles of carbamazepine and sodium alginate were obtained by first compressing the powdered samples in a small cell at 1347 bar (Carver manual hydraulic press, model B, Carver, Inc., Wabash, IN, USA) for 1 h. Contact angles of the individually compressed solutes were then measured using the sessile drop techniques, as previously explained in Section 2.4.

Membrane–carbamazepine as well as membrane–sodium alginate interaction energies were calculated from the surface tension components by solving Equations (13) and (14) using Solver MS Excel, where m and s represent the membrane and solute interacting in liquid, l:

(13) $$∆G_{slm}^{LW}=2(\sqrt{\gamma_{l}^{LW}}-\sqrt{\gamma_{s}^{LW}})(\sqrt{\gamma_{m}^{LW}}-\sqrt{\gamma_{l}^{LW}})$$

(14) $$∆G_{slm}^{AB}=2\sqrt{\gamma_{l}^{+}}\left(\sqrt{\gamma_{s}^{-}}+\sqrt{\gamma_{m}^{-}}-\sqrt{\gamma_{l}^{-}}\right)+2\sqrt{\gamma_{l}^{-}}(\sqrt{\gamma_{s}^{+}}+\sqrt{\gamma_{m}^{+}}-\sqrt{\gamma_{l}^{+}})-2\sqrt{\gamma_{s}^{+}\gamma_{m}^{-}}-2\sqrt{\gamma_{s}^{-}\gamma_{m}^{+}}$$

The total free energy of adhesion ($∆G_{slm}^{TOT}$) between the membranes and solutes in water was quantified from Equation (15).

(15) $$∆G_{slm}^{TOT}=∆G_{slm}^{LW}+∆G_{slm}^{AB}$$

## 3. Results and Discussion

### 3.1. Characteristics of Nanoparticles

Graphene oxide (GO) sheets were observed under a scanning electron microscope (Figure S1A). The sheets appeared layered/stacked on top of each other, and this could be attributed to the high affinity of the GO sheets toward each other. The magnification and resolution used in our characterization did not show the clear geometry of the nanoparticles, but the EDS spectra illustrated and confirmed the composition of the prepared materials, as shown in Figure S1F.

According to the dynamic light scattering (DLS) results, the nanoparticles had mean sizes smaller than 500 nm (Table 2). The electrophoretic mobility results revealed that Ag and ZnO nanoparticles had a positive zeta potential at neutral pH. Contrarily, GO, Ag-GO and ZnO-GO nanoparticles were negatively charged, with GO bearing the highest negative zeta potential. The GO had a dominant influence on the measured charge of the composite materials. The presence of Ag and ZnO reduced the zeta potential of the nanohybrids due to charge neutralization.

### 3.2. Characteristics of the Nano-Engineered Membranes

**Figure 2:**
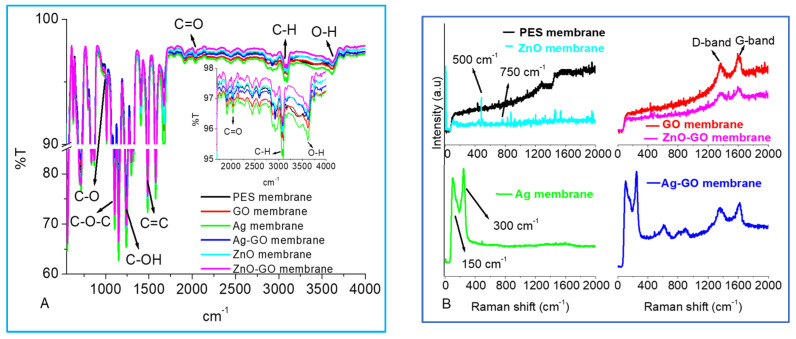
FTIR and Raman spectra of the different nano-engineered membranes. (**A**) FTIR micrographs and (**B**) Raman spectra.

Figure 2 shows FTIR and Raman spectra of the nano-engineered membranes. The addition of the different nanoparticles did not alter the FTIR characteristic peaks of the polymer (Figure 2A). This confirmed the good structural integrity of the membranes. Noticeable peaks at 620 and 880 cm^−1^ were characteristic of C-stretching and C=C stretching on the aromatic rings, respectively [33]. Prominent peaks at 1150 cm^−1^, 1240 cm^−1^ and 1481 cm^−1^ were due to the presence of sulfonyl groups, which is characteristic of PES. The peak at 1244 cm^−1^ was due to the aromatic ether (C–O–C) group. These observations are consistent with previous reports [47].

Raman spectra of the modified membranes showed prominent D-bands and G-bands for PES membranes modified with GO, Ag-GO and ZnO-GO (Figure 2B). The G-bands were due to the graphitic carbon in the structure, while the D-bands were associated with defects or disordered domains in the graphitic domain [34]. The presence of the G and D bands was ascribed to the first-order scattering from the E2g phonon of sp^2^ carbon atoms [48]. The band between 300 cm^−1^ and 400 cm^−1^ was due to the presence of ZnO crystals [35]. The peaks symbolizing the presence of Ag and ZnO nanoparticles were overshadowed when the nanoparticles were incorporated into the membrane polymer matrix. This could be due to the lower concentrations of the nanoparticles used to avoid potential clogging of the membrane pores resulting in lower fluxes [10]. The Raman results confirmed the successful incorporation of the nanoparticles, while the FTIR results showed that the chemical composition of the membranes was not changed to a greater extent.

**Figure 3:**
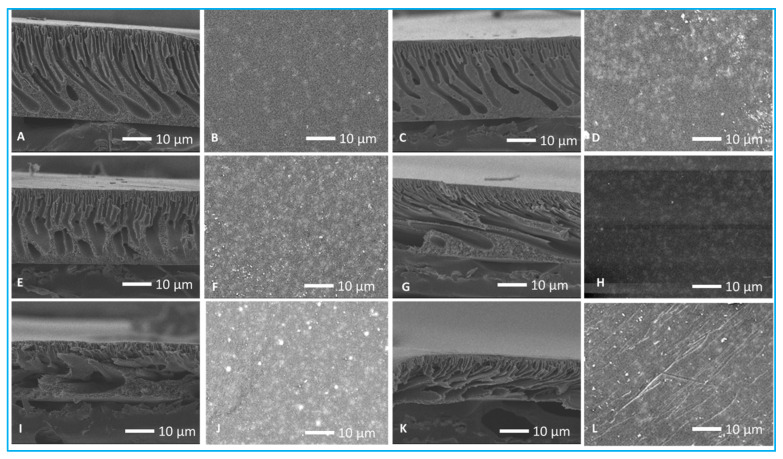
SEM surface and cross-section micrographs of nano-engineered membranes. (**A**,**B**) PES membrane; (**C**,**D**) GO membrane; (**E**,**F**) Ag membrane; (**G**,**H**) Ag-GO membrane; (**I**,**J**) ZnO membrane; (**K**,**L**) ZnO-GO membrane.

The effects of the addition of nanoparticles on the membrane surface and cross-sectional morphology were examined using SEM analysis. The surface of the pristine PES membrane (Figure 3B) appeared smoother than the micrographs of the PES membranes modified with nanoparticles (Figure 3D,F,H,J,L). However, SEM cannot be used to estimate membrane surface roughness; thus, AFM analysis was performed.

The PES membranes were characterized by fingerlike microvoids (Figure 3A,C,E,G,I,K), which portrayed strong interactions between the filler and polymer (PES). These interactions include the formation of C–C bonds between the nanoparticles and PES backbones, as well as hydrogen bonds between the joined groups and water molecules [49]. These microvoids were smaller in the skin layer. For the membranes incorporated with nanoparticles (Figure 3C,E,G,I,K), the microvoids appeared larger than those of the pristine membrane. This can be attributed to the delay in the onset of water–NMP demixing due to the presence of nanoparticles leading to the formation of larger microvoids. Further, the presence of polar groups in the nanoparticles disordered the polymer chains and increased the entropy of the membranes. The resultant effect was an increase in the membrane surface roughness and the widening of the pore diameter [49]. The addition of inorganic nanoparticles also changed the dope solution’s physical properties, such as viscosity, which influenced NMP–water exchange rates. This increase in the size of microvoids was expected to enhance membrane water flux since they present a less torturous path for water transport (less resistant path).

**Figure 4:**
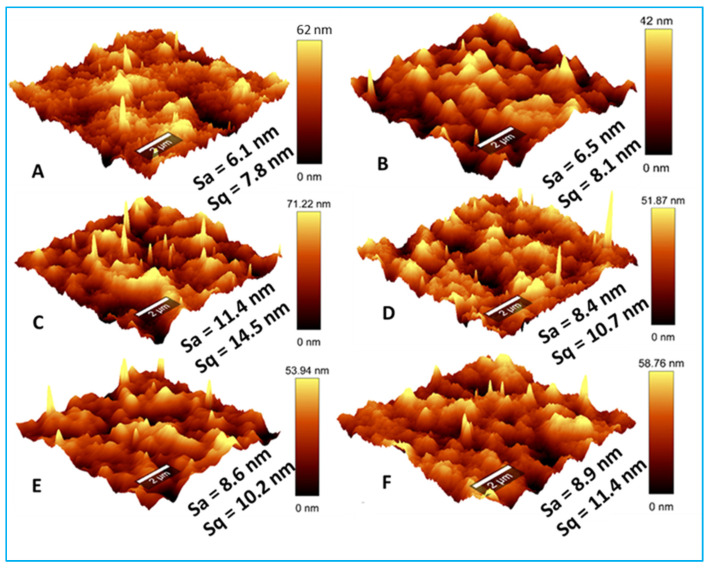
AFM micrographs of the nano-engineered membranes. (**A**) PES membrane; (**B**) GO membrane; (**C**) Ag membrane; (**D**) Ag-GO membrane; (**E**) ZnO membrane; (**F**) ZnO-GO membrane.

In addition to surface imaging using SEM, an atomic force microscope was utilized to probe modifications in the surface roughness of the membranes due to the addition of nanoparticles (Figure 4). Control FOUR software (WITec, GmbH, Germany) was used to quantify the average arithmetic roughness (S_a_) and the root mean square roughness (S_q_), which represent membrane roughness parameters. The general observation from the presented results was that the prepared membranes were generally smooth with S_a_ values lower than 12 nm. Though a closer analysis revealed that the roughness of the membranes increased upon the addition of nanoparticles, signifying an enlargement in the effective membrane surface area. The previously noted increase in microvoids (SEM characterization), as well as the increase in effective membrane surface area (AFM characterization), was expected to enhance membrane water flux [50].

### 3.3. Membrane Flux, Hydrophobicity, Zeta Potential, Salt Removal and Surface Tension Parameters

Membrane characteristics, including wettability, pure water permeability, zeta potential and salt rejection, are presented in Table 3, while membrane surface tension components are presented in Table 4. The control PES membrane was slightly hydrophilic with a contact angle of 77 ± 2°. The addition of nanoparticles improved membrane surface hydrophilicity as observed by the decrease in the water contact angle, and these results are consistent with the literature findings, which have attributed this observation to the introduction of oxygen-rich functional groups [10,51,52]. Further, the PES membranes became more hydrophilic upon adding nanoparticles due to the slight increase in surface roughness (Figure 4), as explained by the Wenzel equation (${cos\theta }_{m}=r{cos\theta }_{\gamma }$), where $\theta_{m}$ is the measured contact angle and $\theta_{\gamma }$ is the Young contact angle [53]. The increase in membrane hydrophilicity resulted in an improvement in the pure water permeability of the membranes. The increase in water permeability could also be attributed to the increase in membrane microvoids (Figure 3), surface roughness (Figure 4) and mean pore radius (Table 3). The removal of salts (MgSO_4_) was poor (<10%), and this was expected because the PES polymer is well known to poorly reject salts [54]. The removal of MgSO_4_ was believed to be through charge interactions, where the interplay between the fixed charges on the PES membranes and the mobile salt might have promoted the removal of MgSO_4_. At pH 7, the PES membranes were negatively charged (Table 3) due to the deprotonation of the functional groups of the PES polymer (-SO_3_H) and nanoparticles (epoxy, hydroxyl and carboxylic functional groups) [55]. Mg^2+^ and SO_4_^2−^ species were removed because of repulsive charge interactions between the membranes and the SO_4_^2−^ ions. Subsequently, Mg^2+^ was rejected to maintain electrical neutrality on the membrane surface; otherwise, a potential difference would be created [56]. High salt removal properties were not the major desired characteristics because the membranes were targeted for application in wastewater treatment, which normally contains low salt concentrations. In addition, the presence of the nanoparticles was aimed at inducing additional membrane removal mechanisms, such as polar and non-polar interactions.

Surface tension components of the pristine and nano-enabled membranes were computed from the measured contact angles as previously explained, and the results are presented in Table 4. The main contributor to the total surface tension component (γ^Total^) was the Lifshitz–van der Waals components (γ^LW^). It was noted that the Lewis base or electron donor components (γ^−^) were higher than the counterpart Lewis acid or electron acceptor components (γ^+^), and this observation was consistent for all membranes. The γ^−^ increased distinctively in all membranes upon the addition of nanoparticles, and this indicated that the membranes became more polar, which correlated with the measured water contact angles (Table 3). It was further noted that γ^LW^ for Ag membranes and ZnO membranes were higher than those of Ag-GO membranes and ZnO-GO membranes. This showed that the presence of GO reduced the Lifshitz–van der Waals components while increasing the electron donor components (γ^−^) due to the presence of oxygen-rich functional groups.

The extensively characterized membranes were investigated for their efficiency in removing carbamazepine and the prevention of organic fouling by sodium alginate. The next sections present the rejection and fouling propensity of the nano-engineered membranes, where the roles of membrane–solute affinity interactions in carbamazepine retention and membrane fouling were investigated.

### 3.4. Carbamazepine Rejection and Organic Fouling Propensity

**Figure 5:**
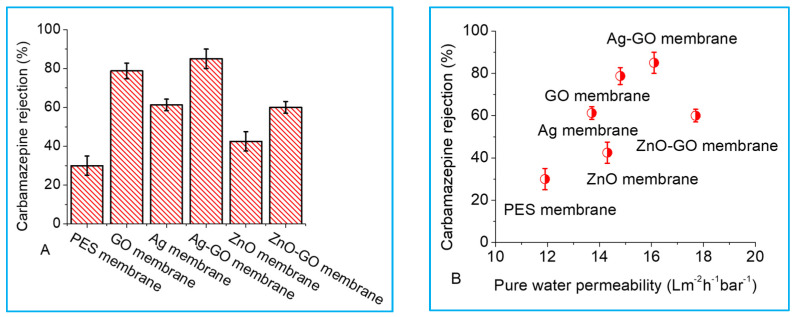
Carbamazepine rejection (**A**) and role of membrane pure water permeability in carbamazepine rejection (**B**) for the nano-engineered membranes. Experimental conditions: 5 mg/L carbamazepine, 10 mM NaCl, pH 6.8, initial flux of 40 L/m^2^ h and crossflow velocity of 0.2 m/s.

The control PES membrane rejected about 30% carbamazepine (Figure 5A), and the addition of nanoparticles improved carbamazepine rejection. This was more apparent for the GO membrane and Ag-GO membrane where carbamazepine rejection ≥80% was achieved. Carbamazepine rejection by Ag and ZnO membranes was lower than that of Ag-GO as well as ZnO-GO membranes. This showed the synergistic effects of the different materials in improving membrane separation qualities for more effective performance. Further, the improvement could be linked to the increase in electron donor components (γ^−^), which were expected to lower solute affinity for the membrane surface, thus improving rejection. Ag-GO and ZnO-GO membranes had the highest electron donor components (Table 4).

The rejection of organic compounds is controlled by both the membrane and solute properties, where the major rejection mechanisms are size exclusion, electrostatic interactions and non-electrostatic interactions, which include hydrophobic interactions and the formation of hydrogen bonds [9]. Carbamazepine is a neutral compound; therefore, no charge interactions with the membrane were expected. All the membranes had a mean pore radius of less than 5 nm with the pristine membrane having the lowest mean pore radius of 2.39 nm (Table 2). Based on the mean pore size of the membranes, carbamazepine was rejected through size exclusion, but this was not the only mechanism for carbamazepine removal, as the membranes modified with nanoparticles had larger pores (implying a high molecular weight cut-off) but still achieved higher carbamazepine retention than the pristine membrane. Carbamazepine is a transphilic organic solute with a log K_ow_ value of 2.45 [57]. Previous studies have reported that the rejection of organic compounds is also controlled by membrane–solute hydrophobic interactions, where hydrophobic membranes show lower solute rejection due to the adsorption of the compounds onto the membrane surface and facilitate diffusion into the permeate side, leading to lower rejection [9,13,58]. There was a clear increase in carbamazepine rejection with the increase in membrane hydrophilicity (due to the addition of nanoparticles). This increase was expected because a water layer formed on the vicinity (interface) of the surfaces of the more hydrophilic membranes, and this reduced membrane–solute interactions due to overlapping hydration layers leading to high solute rejection [59]. The addition of nanomaterials is believed to have introduced defects into the PES, and the superficial imperfections provided interfacial areas that are covered by water droplets during filtration [60,61].

Other studies have reported that the rejection of organics by dense membranes improved with an increase in permeate flux, and this was attributed to the dilution effects [27,29,30]. To relate rejection to membrane pure water permeability, carbamazepine rejection was plotted as a function of pure water permeability (Figure 5B), and it was noted that more carbamazepine was rejected with the increase in membrane pure water permeability. These results showed that the more permeable the membranes were to pure water, the more solutes were rejected. However, the trend was not very clear because the performance of the fabricated membranes depends mainly on the pore size and pore size distribution, porosity and morphological structure as well as the charge of the membrane and solute [20]. There is a tradeoff between water permeability and solute rejection properties, which implies that water permeability and rejection cannot be maximized at the same time [9,31]. At a working polymer/nanoparticle mass ratio, both permeability and rejection were improved without compromising either of the two.

**Figure 6:**
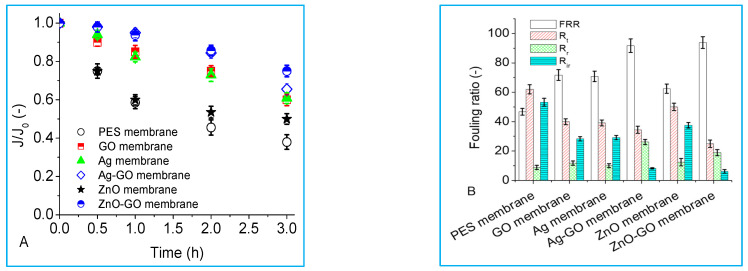
Membrane fouling (flux decline) by sodium alginate and fouling resistance parameters. (**A**) Normalized flux decline; (**B**) fouling resistance parameters. Fouling conditions: 20 mg/L sodium alginate, background electrolyte concentration of 10 mM NaCl, pH 6.8, initial flux of 40 L/m^2^ h and crossflow velocity of 0.2 m/s.

The application of membranes is greatly hindered by fouling, which deteriorates both membrane flux and performance in terms of solute rejection. In this study, PES membranes were modified with different nanoparticles to reduce organic fouling (using sodium alginate as a representative of natural organic matter), and the influence of membrane modification with nanoparticles was more evident, as fouling was lower for the nano-engineered membranes compared to the pristine membrane (Figure 6A). The flux declined the least for the membranes modified with hybrid Ag-GO and ZnO-GO nanoparticles, showing synergistic effects in improving antifouling properties by the nanomaterials. A similar observation was made for carbamazepine rejection, where PES membranes with combined nanohybrids rejected the organic compound more than PES membranes with individual nanoparticles. This was attributed to the Ag-GO and ZnO-GO membranes having more polar groups or electron donor components ($\gamma^{-}$, Table 4).

Fouling is controlled by an interplay between membrane properties, foulant properties and the chemistry of the feed solution. The feed water chemistry and foulant properties were the same for all fouling experiments; thus, the differences in fouling extent could not be due to the variation in feed chemistry and the properties of the foulants. The characterization results revealed that the sodium alginate had a particle size of 125 ± 1.6 nm and a zeta potential of −34.9 ± 3.2 mV. Sodium alginate was larger than the surface pore radius of the membranes (Table 3). Therefore, no foulants were expected to penetrate the membrane pores, resulting in complete pore blocking. Fouling was promoted by permeation drag and non-electrostatic interactions between sodium alginate and the membranes. At a working pH of 7, the membranes and foulants were negatively charged; therefore, fouling was not anticipated to occur due to repulsive charge interactions. However, since fouling was observed, this could be linked to other factors, such as permeation drag and non-electrostatic interactions, such as hydrophobic interactions [36]. The contact angle results (Table 3) showed that the PES membrane was the least hydrophilic, with a contact angle of 77 ± 2°, which made it slightly hydrophobic, while the Ag-GO membrane was the most hydrophilic (62 ± 2°). The PES membrane fouled more due to the favored adsorption of sodium alginate onto the membrane surface [10,13,15]. Thus, the addition of nanoparticles improved the ability of membranes to resist organic fouling by lowering solute affinity for the membrane surface. An increase in membrane surface roughness was noted upon the addition of nanoparticles. Ideally, high surface roughness provides attachment sites, and during filtration, the “valleys” on the membrane zone present dead zones free of the cross-sectional shear force and are ideal for foulant adhesion. However, this was not observed for the nano-engineered membranes because the increase in membrane hydrophilicity favored the formation of hydration layers, which reduced foulant interactions with the membrane surfaces [59,60].

Membrane fouling could be temporal (reversible—requires hydraulic cleaning) or permanent (irreversible—requires chemical cleaning). The total fouling ratio ($R_{t}$) is a combination of the reversible fouling ratio ($R_{r}$) and irreversible fouling ratio ($R_{ir}$). To understand the antifouling properties of the membranes, the fouling ratios were quantified, and it was found that the major contributor to flux decline for the control PES membrane was irreversible fouling (Figure 6B). The addition of nanoparticles reduced the total fouling ratio ($R_{t}$) by decreasing irreversible fouling through inhibiting hydrophobic membrane-foulant affinity interactions. Up to 90% flux recovery was achieved for the Ag-GO and ZnO-GO membranes, while there was less than 50% flux recovery from the control PES membrane after flushing with deionized water, and this was due to the permanent fouling of the membrane. 

### 3.5. Role of Membrane–Solute Interaction Energies on Solute Rejection and Membrane Fouling

**Figure 7:**
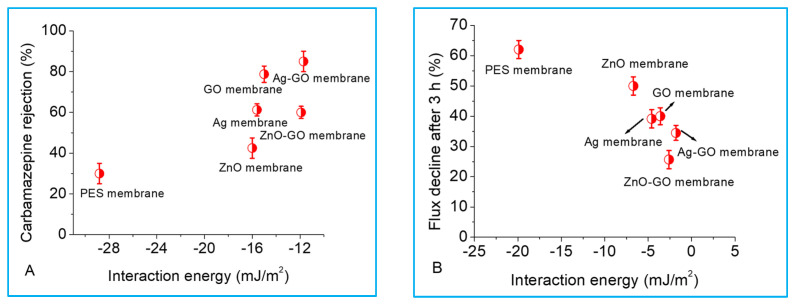
Role of interaction energies in carbamazepine rejection (**A**) and flux decline during sodium alginate fouling (**B**). Experimental conditions: 5 mg/L carbamazepine, 10 mM NaCl, pH 6.8, initial flux of 40 L/m^2^ h and crossflow velocity of 0.2 m/s. In fouling experiments, 20 mg/L sodium alginate was used.

In addition to size exclusion and charge interactions, the rejection of organic compounds is also influenced by non-electrostatic membrane–solute affinity interplay. In this study, membrane–solute affinity interactions or free energies of adhesion (${∆G}_{slm}$) were predicted from the contact angle measurements and related to carbamazepine rejection, as well as flux decline. A positive interaction energy implies repulsive forces between the solute (*s*) and membrane (*m*) in a liquid medium (l), while a negative interaction energy implies attractive interactions. Carbamazepine rejection improved as the membrane–solute affinity became less attractive (Figure 7A). Therefore, there was poor contact between carbamazepine and the membrane surface, thus minimizing carbamazepine adsorption onto the membrane surface. These findings are in agreement with previous results that have shown the resistance of organics against partitioning into the membrane phase as ${∆G}_{slm}$ became more positive [10,11,27]. However, the works of Ma et al. [11] and that of Mahlangu et al. [27] studied solute rejection using well-characterized commercial ion exchange (AEM I, CEM I, AEM II and CEM II) and nanofiltration (NF270) membranes, respectively. Our work shows that affinity interactions are also important for nano-engineered membranes and need to be characterized fully if these membranes are to be commercialized.

In the early stages of fouling, the deposition of foulants on the membrane surface is controlled by membrane–foulant interactions, in this case, PES/modified PES membranes and sodium alginate. Once the membrane surface is completely covered by foulants, subsequent fouling is then dominated by foulant–foulant interplay, which is completely independent of the membrane properties [36]. The aim of modifying the membrane surface properties is to minimize foulant affinity for the membrane surface by making (${∆G}_{slm}$) more positive (repulsive). Therefore, in this study, the role of membrane–foulant adhesion energies in fouling or foulant deposition on the membrane surface was investigated by plotting flux decline as a function of ${∆G}_{slm}$. Again, there was low fouling when adhesion energies were less attractive (Figure 7B), showing that initial fouling was also influenced by membrane–foulant adhesion energies, thereby agreeing with findings from the works of Jin et al. who studied the fouling of a seawater reverse osmosis membrane by alginic acid [28]. Our results show that affinity interactions are important regardless of the membrane type and salt rejection properties of the membrane.

**Figure 8:**
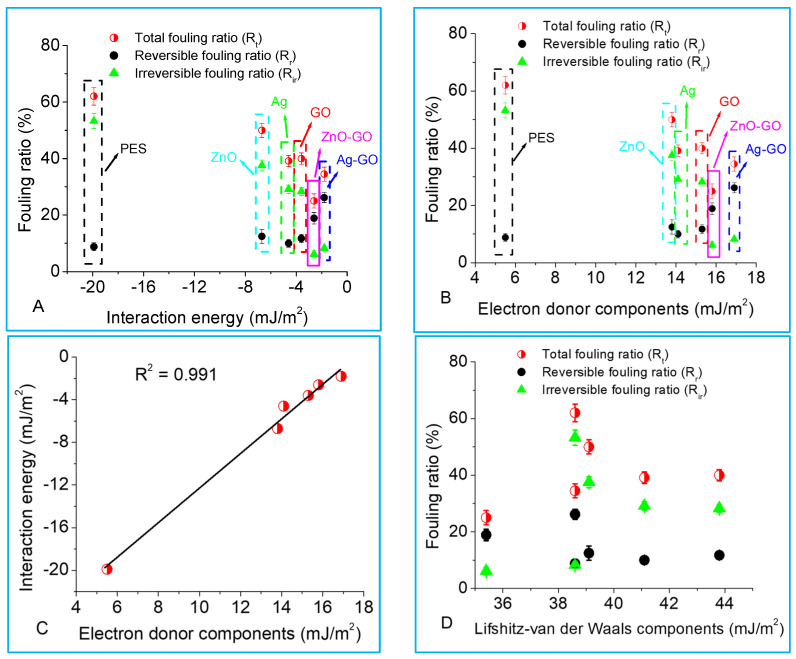
Relation between interaction energies and membrane fouling ratios (total fouling (Rt), reversible fouling (Rr) and irreversible fouling (Rir) ratios). (**A**) Relation between membrane–sodium alginate adhesion forces and fouling ratios; (**B**) influence of electron donor components ($\gamma^{-}$) on fouling ratios; (**C**) correlation between electron donor components and interaction energy; and (**D**) influence of Lifshitz–van der Waals components ($\gamma^{LW}$) on fouling ratios.

To study the link between fouling parameters and ${∆G}_{slm}$, the quantified fouling ratios were uniquely plotted as a function of membrane–foulant adhesion energy (Figure 8A). It was observed that the total fouling ratio ($R_{t}$) as well as the irreversible fouling ratio ($R_{ir}$) declined with an increase in $∆G_{slm}$ (due to membrane modification with nanoparticles), while the reversible fouling ratio ($R_{r}$) increased. The PES membrane was characterized by a high total fouling ratio, where irreversible fouling contributed the most to flux decline. However, this was reduced upon adding nanoparticles, and fouling was mainly reversible (high flux recovery ratio, Figure 6B). Reversible fouling occurred due to hydraulic resistance and permeation drag, but there was probably no formation of permanent bonds between the membranes and foulants. The higher flux recovery for Ag-GO and ZnO-GO membranes was due to the reversible fouling contributing more to the total fouling ratio (Figure 8A,B). Fouling was reversible due to the membranes having more electron donor components (Table 4), and this made membrane–foulant interactions repulsive (i.e., less negative, Figure 7B).

Membrane total interfacial parameters comprise $\gamma^{LW}$, and $\gamma^{-}$ and $\gamma^{+}$. To gain further insights into the membrane surface tension components that control fouling, the fouling ratios were plotted as a function of $\gamma^{LW}$, $\gamma^{-}$ and $\gamma^{+}$, and it was found that fouling became more reversible with an increase in $\gamma^{-}$ (Figure 8B). This is because $∆G_{slm}$ increased with electron donor components ($\gamma^{-}$) in a linear relationship (Figure 8C). This further confirmed that adding more electron-withdrawing groups to the membrane polymer improves the rejection and antifouling properties. Previous studies have ascribed this observation to membranes becoming more hydrophilic—our work details this to be due to the reduction in membrane–solute affinity interactions. There was no correlation between the different fouling ratios neither with Lifshitz–van der Waals components (Figure 8D) nor the electron acceptor components (Figure S2).

Membrane fouling was confirmed using SEM and a Spotlight 400 Imaging system. The PES membrane had the most foulants deposited on the surface (Figure S3A,B), and the fouling layer was clearly visible to the naked eye. In general, there was less deposition of foulants on the surfaces of membranes modified with nanoparticles, and the membranes incorporated with combined nanoparticles showed minimal foulant build-up (Figure S3C–L). The lesser accumulation of the foulant for nano-engineered membranes was due to the reduced membrane–foulant affinities (${∆G}_{slm}$), which could be attributed to the membranes gaining more electron donor components ($\gamma^{-}$) upon the incorporation of oxygen-rich nanoparticles (Table 3).

## 4. Conclusions

Polyethersulfone (PES) membranes were modified with various nanoparticles to enhance the rejection and antifouling properties. The incorporation of nanoparticles improved the membrane properties, such as tensile strength (3.46–4.11 N/mm^2^), hydrophilicity (77–62°) and pure water permeability (11.9–17.7 Lm^−2^ h^−1^ bar^−1^). Further, nanoparticle addition increased the electron donor components of the membranes, which improved the rejection of carbamazepine (30–>80%) and antifouling properties (a reduction in fouling from 60 to 23%). Rejection and fouling prevention were enhanced due to the decrease in solute affinity for the membrane surface. This ensured low/minimal adsorption of the solutes (both organic pollutants and foulants) on the membrane surface. Incorporating nanoparticles into PES membranes changed the fouling pattern from irreversible fouling to reversible fouling, where the fouled membranes were cleaned by flushing with water to recover flux. Solute rejection and fouling were found to be influenced by electron donor components ($\gamma^{-}$) more than Lifshitz–van der Waals ($\gamma^{LW}$) and electron acceptor components ($\gamma^{+}$). These results show that membranes incorporated with nanoparticles have the potential to achieve high removal of organic contaminants in wastewater while retaining high fluxes due to minimum fouling. This translates to energy saving by nano-engineered membranes. However, the stability of the nanoparticles over long-term applications needs to be investigated, as some studies have hinted at the potential release of nanoparticles from polymeric membranes. Membrane fouling can be alleviated by adding electron donor components through membrane impregnation with oxygen-rich nanomaterials. This also improves the removal of organic compounds.

## Figures and Tables

**Table 1:** Composition of casting solutions.

Membrane Name	Concentration (wt%)				
	PES	NMP	Nanoparticle		
			GO	Ag	ZnO
PES membrane	22	78.0	0	0	0
GO membrane	22	77.8	0.2	0	0
Ag membrane	22	77.8	0	0.2	0
Ag-GO membrane	22	77.8	0.1	0.1	0
ZnO membrane	22	77.8	0	0	0.2
ZnO-GO membrane	22	77.8	0.1	0	0.1

**Table 2:** Particle size and zeta potential of the fabricated nanomaterial at neutral pH and 10 mM KCl background electrolyte.

	Size (nm)	Zeta Potential (mV)
GO	220.2 ± 72	−22.4 ± 1.2
Ag	98 ± 17	25.6 ± 0.9
Ag-GO	382 ± 30	−4.7 ± 0.2
ZnO	392.4 ± 55	28.8 ± 1.8
ZnO-GO	420.5 ± 83	−6.9 ± 0.7

**Table 3:** Water contact angle, pure water permeability (PWP), zeta potential, tensile strength, magnesium rejection and mean pore radius for the nano-engineered membranes.

	Contact Angle (°)	PWP (L/m^2^ hbar)	Zeta Potential (mV)	Stress (N/mm^2^)	MgSO_4_ Rejection (%)	Mean Pore Radius (nm)
PES membrane	77 ± 2	11.9 ± 1.5	−29.8 ± 2	3.46 ± 0.3	8.5 ± 0.2	23.9 ± 1.1
GO membrane	65 ± 2	14.8 ± 1.2	−25.9 ± 1	3.79 ± 0.5	8.3 ± 0.3	31.8 ± 2.3
Ag membrane	68 ± 2	13.7 ± 0.8	−24.2 ± 1	2.69 ± 0.2	8.7 ± 0.1	27.8 ± 2.5
Ag-GO membrane	62 ± 2	16.1 ± 1.8	−23.4 ± 2	3.89 ± 0.5	8.6 ± 0.2	44.1 ± 2.1
ZnO membrane	63 ± 2	14.3 ± 1.3	−25.9 ± 3	2.11 ± 0.3	9.1 ± 0.2	37.7 ± 1.4
ZnO-GO membrane	65 ± 2	17.7 ± 1.7	−23.5 ± 2	4.11 ± 0.6	9.5 ± 0.3	48.4 ± 2.3

**Table 4:** Surface tension parameters of nanocomposite membranes.

	Surface Tension Parameters (mJ/m^2^)			
	$\gamma^{LW}$	$\gamma^{+}$	$\gamma^{-}$	$\gamma^{Total}$
PES membrane	38.6 ± 4	0.6 ± 0.1	5.5 ± 1	42.1 ± 3
GO membrane	43.8 ± 3	0.1	15.3 ± 2	45.5 ± 4
Ag membrane	41.1± 4	0.1	14.1 ± 2	42.8 ± 3
Ag-GO membrane	38.6 ± 2	0.5 ± 0.1	16.9 ± 3	44.6 ± 2
ZnO membrane	39.1 ± 3	0.9 ± 0.1	13.8 ± 2	46.4 ± 3
ZnO-GO membrane	35.4 ± 2	0.6 ± 0.1	15.8 ± 2	41.6 ± 2

## Data Availability

All data reported in this work are available upon request.

## Supplementary Materials

The following supporting information can be downloaded at: https://www.mdpi.com/article/10.3390/membranes13080744/s1, Figure S1: Scanning electron micrographs (SEMs) and EDS spectra of the synthesized material. A—GO nanoparticles, B—Ag nanoparticles, C—Ag-GO nanoparticles, D—ZnO nanoparticles, E—ZnO-GO nanoparticles and F—combined EDS spectra of the nanoparticles; Figure S2: Relationship between membrane fouling ratio and electron acceptor components ($\gamma^{+})$ for membrane fouling with sodium alginate; Figure S3: SEM and microscope images of the polymeric membranes after fouling with 20 mg/L sodium alginate: A–B—PES membrane; C–D—GO membrane; E–F—Ag membrane; G–H—Ag-GO membrane; I–J—ZnO membrane; K–L—ZnO-GO membrane.
