# Supplementary material for: Role of Membrane–Solute Affinity Interactions in Carbamazepine Rejection and Resistance to Organic Fouling by Nano-Engineered UF/PES Membranes
Source: Membranes (Basel). 2023 Aug 21;13(8):744. doi: 10.3390/membranes13080744 (PMC10456577; doi:10.3390/membranes13080744)
Supplement: Supplementary file 1 [file membranes-13-00744-s001.zip › membranes-2542291-supplementary.pdf]

membranes

Article

Role of Membrane–Solute Affinity Interactions in Carbamazepine Rejection and Resistance to Organic Fouling by Nano-Engineered UF/PES Membranes

Oranso Themba Mahlangu [1,2,*], Mxolisi Machawe Motsa [1], Faisal Ibney Hai [2] and Bhekie Brilliance Mamba [1]

[1] Institute for Nanotechnology and Water Sustainability, College of Engineering, Science and Technology, University of South Africa, Florida Science Campus, Roodepoort 1709, South Africa; motsamm@unisa.ac.za (M.M.M.); mambabb@unisa.ac.za (B.B.M.)
[2] Strategic Water Infrastructure Laboratory, School of Civil, Mining and Environmental Engineering, University of Wollongong, Wollongong, NSW 2522, Australia; faisal@uow.edu.au
* Correspondence: mahlaot@unisa.ac.za

Supporting Information

Graphene oxide (GO) sheets were observed under a scanning electron microscope (Fig. S1A). The sheets appeared layered/stacked on top of another, and this could be attributed to the high affinity of the GO sheets towards each other. The magnification and resolution used in our characterization did not show the clear geometry of the nanoparticles (due to instrument limitation), but the EDS spectra illustrate and confirm the composition of the prepared materials as shown in Fig S1F. The GO sheets were more observable in the ZnO-GO hybrid. EDS spectra showed the following chemical composition and confirmed successful synthesis of the nanoparticles: GO nanoparticles – carbon and oxygen; Ag nanoparticles – silver; Ag-GO nanoparticles – silver, carbon, and oxygen; ZnO nanoparticles – zinc and oxygen and ZnO-GO – zinc, oxygen, and carbon (Fig. S1F).

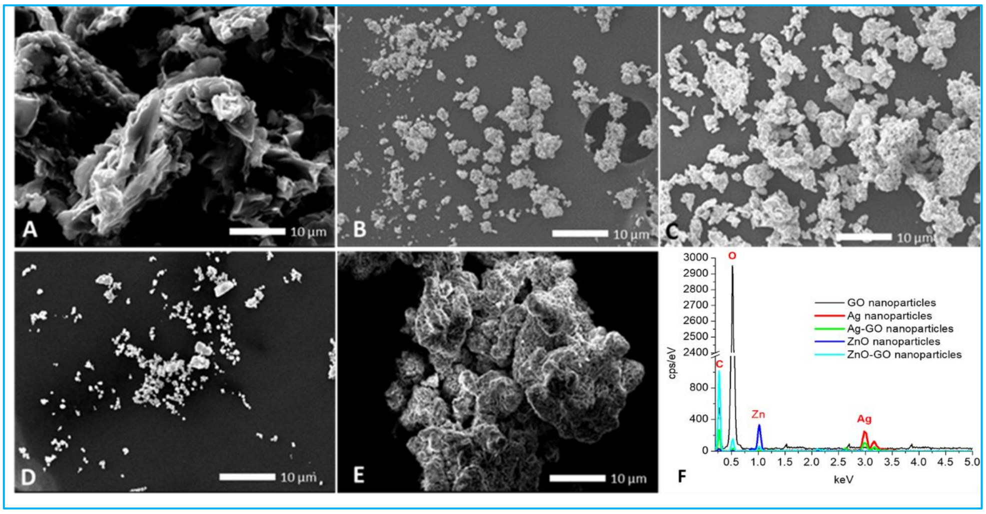

Figure S1. Scanning electron micrographs (SEM) and EDS spectra of the synthesized material. A – GO nanoparticles, B – Ag nanoparticles, C – Ag-GO nanoparticles, D – ZnO nanoparticles, E – ZnO-GO nanoparticles and F – combined EDS spectra of the nanoparticles.

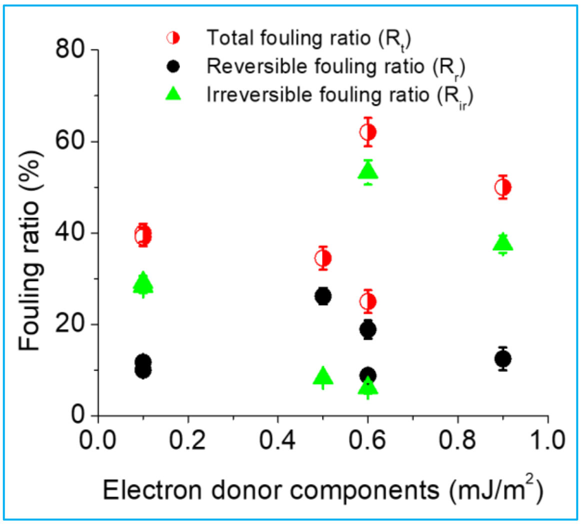

Figure S2. Relation between membrane fouling ratio electron acceptor components (γ^+) for membrane fouling sodium alginate.

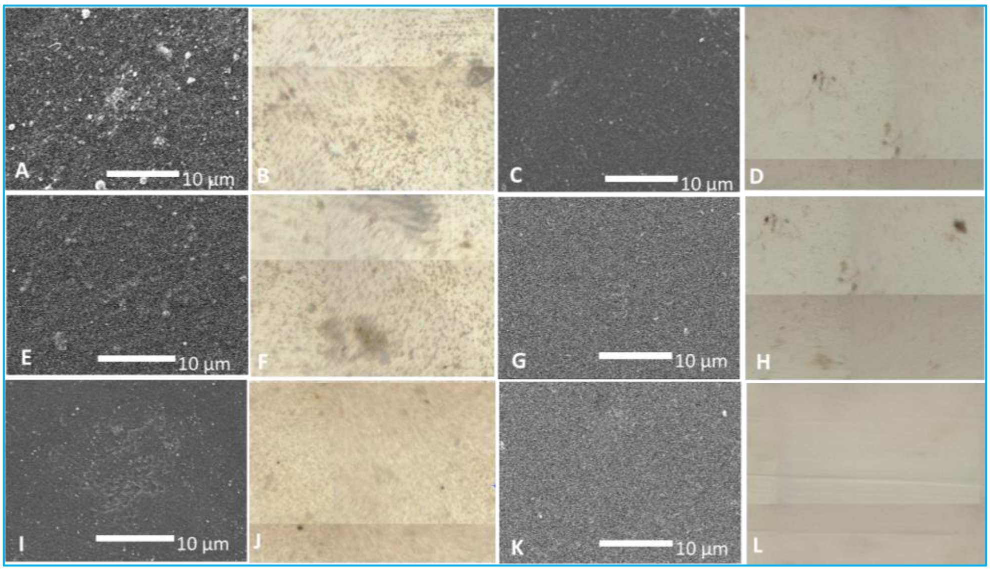

Figure S3. SEM micrographs and microscope images of the polymeric membranes after fouling with 20 mg/L sodium alginate: A&B – PES membrane; C&D – GO membrane; E&F – Ag membrane; G&H – Ag-GO membrane; I&J – ZnO membrane; K&L – ZnO-GO membrane.
